# SetBERT: the deep learning platform for contextualized embeddings and explainable predictions from high-throughput sequencing

**DOI:** 10.1093/bioinformatics/btaf370

**Published:** 2025-06-25

**Authors:** David W Ludwig, Christopher Guptil, Nicholas R Alexander, Kateryna Zhalnina, Edi M -L Wipf, Albina Khasanova, Nicholas A Barber, Wesley Swingley, Donald M Walker, Joshua L Phillips

**Affiliations:** Department of Computer Science, Middle Tennessee State University, Murfreesboro, TN 37132, United States; Department of Mathematics and Computer Science, Miami University, Oxford, OH 45056, United States; Department of Biology, Middle Tennessee State University, Murfreesboro, TN 37132, United States; Environmental Genomics and Systems Biology, Lawrence Berkeley National Laboratory, Berkeley, CA 94720, United States; Environmental Genomics and Systems Biology, Lawrence Berkeley National Laboratory, Berkeley, CA 94720, United States; Environmental Genomics and Systems Biology, Lawrence Berkeley National Laboratory, Berkeley, CA 94720, United States; Department of Biology, San Diego State University, San Diego, CA 92182, United States; Department of Biological Sciences, Northern Illinois University, DeKalb, IL 60115, United States; Department of Biology, Middle Tennessee State University, Murfreesboro, TN 37132, United States; Department of Computer Science, Middle Tennessee State University, Murfreesboro, TN 37132, United States

## Abstract

**Motivation:**

High-throughput sequencing (HTS) is a modern sequencing technology used to profile microbiomes by sequencing thousands of short genomic fragments from the microorganisms within a given sample. This technology presents a unique opportunity for artificial intelligence to comprehend the underlying functional relationships of microbial communities. However, due to the unstructured nature of HTS data, nearly all computational models are limited to processing DNA sequences individually. This limitation causes them to miss out on key interactions between microorganisms, significantly hindering our understanding of how these interactions influence the microbial communities as a whole. Furthermore, most computational methods rely on post-processing of samples which could inadvertently introduce unintentional protocol-specific bias.

**Results:**

Addressing these concerns, we present SetBERT, a robust pre-training methodology for creating generalized deep learning models for processing HTS data to produce contextualized embeddings and be fine-tuned for downstream tasks with explainable predictions. By leveraging sequence interactions, we show that SetBERT significantly outperforms other models in taxonomic classification with genus-level classification accuracy of 95%. Furthermore, we demonstrate that SetBERT is able to accurately explain its predictions autonomously by confirming the biological-relevance of taxa identified by the model.

**Availability and implementation:**

All source code is available at https://github.com/DLii-Research/setbert. SetBERT may be used through the q2-deepdna QIIME 2 plugin whose source code is available at https://github.com/DLii-Research/q2-deepdna.

## Graphical abstract

**Figure:**
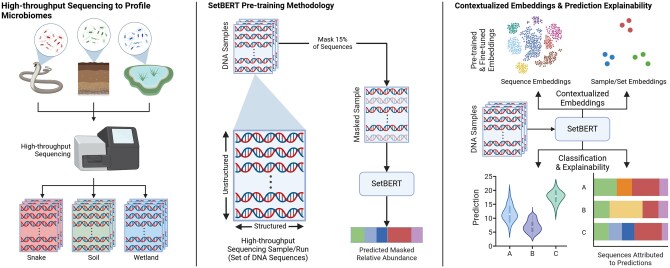

## 1 Introduction

High-throughput sequencing (HTS) is a short-read technology that can rapidly sequence thousands of gene fragments from a single sample. By sequencing gene fragments originating from organisms in a sample or environment, HTS allows scientists to characterize microbial communities comprised mostly of microorganisms that cannot be cultured for identification. Microbial community profiling often employs amplicon sequencing, in which a targeted gene sequence is amplified from samples prior to sequencing. For prokaryotic communities (bacteria and archaea), this often focuses on the 16S rRNA gene, with primers used to amplify specific variable regions of the gene.

Although machine learning has been successful in the field of bioinformatics and computational biology, deep learning shows great potential to push performance even further. Because DNA sequences can be treated analogously to text-based data, many deep learning approaches for processing DNA borrow from natural language processing (NLP) techniques (Ji *et al.* 2021, Mock *et al.* 2021, Ruffolo *et al.* 2021, Nguyen *et al.* 2023, Refahi *et al.* 2023, 2024, Zhou *et al.* 2024a, 2024b). These techniques have also employed pre-training tactics to significantly improve generalization and leverage transfer learning to quickly fine-tune to downstream tasks.

HTS presents a unique opportunity for machine learning and deep learning techniques to go beyond processing single sequences at a time. For example, incorporating abundance/distribution information into machine learning models can significantly improve the model performance (Kaehler *et al.* 2019). However, most models employed in the literature are still limited to processing sequences in isolation (Ji *et al.* 2021, Mock *et al.* 2021, Nguyen *et al.* 2023, Zhou *et al.* 2024a, 2024b). Some recent works employ permutation-equivariant deep learning architectures to process multiple sequences at a time to make more informed predictions. AntiBERTy is pre-trained on single natural antibody sequences and employs multiple-instance learning to cluster the resulting embeddings (Ruffolo *et al.* 2021). SetQuence and SetOmic employ transformers in a supervised-learning manner to classify cancerous whole-genome fragments (Jurenaite *et al.* 2024). A key limitation of these models is that they were designed with a focused application in mind rather than creating a general-use-case model and such approaches have yet to be leveraged for HTS and its associated applications: neither metagenomic sequencing more generally nor amplicon sequencing more specifically. Other common techniques for analysing entire microbiomes such as network analysis rely on knowing the taxa that are present in a sample (Faust 2021, Matchado *et al.* 2021). A general purpose model designed for raw HTS could provide a new and more powerful means to explore and understand the complex interactions within microbial communities.

In this work, we propose SetBERT, an explainable, pre-trainable deep learning architecture and methodology designed with high-throughput amplicon sequencing in mind. SetBERT builds upon the *Set Transformer* (ST) framework (Lee *et al.* 2019) to create an architecture capable of processing thousands of short-read sequences in a permutation-equivariant and sample-context manner. This architecture allows the model to capture the input abundance/distribution information to make contextualized predictions, removing the requirement for specialized models for, or explicit indication of, the biome/habitat. By pre-training SetBERT across a wide variety of biomes/habitats, it can leverage transfer learning to quickly learn downstream tasks and make highly-accurate predictions. Furthermore, unlike the often-assumed black box nature of typical neural network architectures, attention scores from the transformer’s attention mechanism can be mined to find sequences/taxa that have the most predictive impact. These findings have the potential to be used to guide future experiments and inform important experimental variables.

## 2 Materials and methods

### 2.1 Reference sequences and experimental data

This work makes use of reference sequences and taxonomies from the SILVA v138.1 NR99 SSU 16S/18S dataset (Pruesse *et al.* 2007) with the sequences trimmed to the V3–V4 region. We follow the procedures outlined by QIIME 2 to trim the sequences to the V3–V4 region using the standard 515F/806R primer pair (Kozich *et al.* 2013, Quast *et al.* 2013, Bokulich *et al.* 2018, Robeson *et al.* 2021), matching the process of the official QIIME 2 pre-trained taxonomy classifiers. We utilized four experimental amplicon sequencing datasets: Hopland, Nachusa, Snake Fungal Disease (SFD), and Wetland. Full detail regarding the gathering and processing of the samples is provided in the Supplementary Materials, available as supplementary data at *Bioinformatics* online.

### 2.2 The SetBERT architecture

SetBERT is based on the idea of Bi-directional Encoder Representations from Transformers (BERT). BERT is a powerful deep learning model originally designed for NLP to learn contextualized word embeddings (Devlin *et al.* 2019). It is based on the transformer architecture (Vaswani *et al.* 2017), the current state-of-the-art deep learning architecture across many different domains, and employs a pre-training/fine-tuning paradigm. In general, it processes tokenized inputs with a special learned class token [CLS] prepended at the beginning with position embedding information added. The core pre-training objective behind BERT requires the model to reconstruct masked tokens using the contextual clues of the remaining tokens. This process is entirely unsupervised, allowing for the model to be given large amounts of unlabeled data so that it can familiarize itself with the language it is being trained on. Once pre-trained, feeding an input sentence into the model will produce high-quality word embeddings that are contextualized based on the sentence-level information, and the special [CLS] token will be conditioned on the input such that it becomes a contextualized embedding representing the entire sentence.

**Figure 1.**
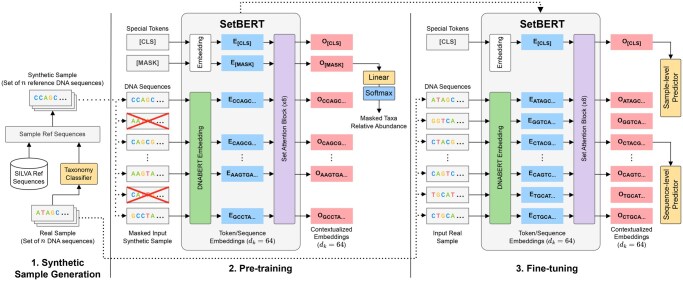
An overview of the SetBERT model. Synthetic samples are first generated following the taxonomy distribution of their corresponding real sample counterparts in order to obtain DNA sequences with ground-truth taxonomy labels. SetBERT is pre-trained on these synthetic samples by inputting 85% of the sample’s DNA sequences along with two special tokens [CLS] and [MASK], where [MASK] is used to predict the relative taxonomy abundance of the missing 15%. The model maps special tokens to learned embeddings, and DNA sequences are embedded with DNABERT (Ji *et al.* 2021) prior to feeding everything through a stack of *set attention blocks* (SABs) (Lee *et al.* 2019) to produce output contextualized embeddings. The output [CLS] embedding comes to represent the entire sample, while the output DNA sequence embeddings represent those sequences “in context” to the sample. After pre-training, SetBERT can be quickly fine-tuned for downstream sample-level and sequence-level modeling tasks using real sample data.

Since the transformer architecture is naturally permutation equivariant, we construct the SetBERT architecture following the methodologies of the ST framework to be compatible with unstructured data (Lee *et al.* 2019). The multi-head attention mechanism within the transformer gates the flow of information by leveraging the interactions in the input. In this work, it is comprised of a stack of eight *set attention blocks* (SABs) (i.e. standard transformer blocks without position information), each with eight attention heads. Figure 1 provides a general overview of the SetBERT model and pipeline. It takes a set of DNA sequences of cardinality *n* as input, where each sequence is drawn from the same sample uniformly with replacement. The learnable [CLS] token (and [MASK] token during pre-training) is prepended to the list of sequences and the tokens are embedded. The [CLS] token and other special tokens are mapped to corresponding learned embeddings. While DNA sequences can be embedded with any kernel of choice, we employ DNABERT (Ji *et al.* 2021), one of the recent state-of-the-art sequence-level embedding models (Wang *et al.* 2023), pre-trained on 150 bp sequences from the SILVA dataset. As we still allow DNABERT’s parameters to be updated during the training of SetBERT, this pre-training serves primarily as a method of bootstrapping SetBERT’s pre-training. The parameter updates will allow DNABERT to fine-tune itself to produce embeddings tailored to SetBERT enabling it to make more effective predictions. The implementation and training details of DNABERT are provided in the Supplementary Materials, available as supplementary data at *Bioinformatics* online. The input special tokens and DNA sequences for SetBERT are embedded to $d_{k}=64$-dimensional vector representations. The embeddings are then passed through the stack of SABs, producing contextualized representations of each input. Each output DNA sequence embedding is a vector representation of that particular DNA sequence in context with the rest of the sample. Likewise, the output [CLS] embedding from SetBERT is a vector representation of the entire sample as a whole after pre-training and/or fine-tuning (Devlin *et al.* 2019, Dosovitskiy *et al.* 2021).

### 2.3 Pre-training SetBERT

While the SABs from the ST framework are enough to make SetBERT capable of processing unstructured data, the traditional BERT pre-training regime is not directly compatible and necessitates modifications. The standard BERT pre-training regime substitutes each of the masked elements with the [MASK] token. The transformer leverages explicitly injected position information to discern one mask token from another. However, in a set context where there is no position information available, the model evaluates each mask token identically, thus the resulting output embedding for each reconstructed element will be identical to every other reconstructed element. To address this challenge, we reworked the BERT objective to instead predict the relative abundance of the masked taxa. Rather than replacing each of the masked sequences with some corresponding mask token, they are all simply removed from input, and a single [MASK] token is appended to the set. After passing everything through the SABs, the mask token is fed through a single linear layer with a softmax activation to predict the normalized relative taxonomy distribution of those taxa that were masked. 

**Table 1.** Feature comparison of models: DNABERT (Ji *et al.* 2021), DNABERT 2 (Zhou *et al.* 2024a), DNABERT-S (Zhou *et al.* 2024b), MetaBERTa (Refahi *et al.* 2023), HyenaDNA (Nguyen *et al.* 2023), SetQuence/SetOmic (Jurenaite *et al.* 2024), glm2 (Cornman *et al.* 2024), Scorpio (Refahi *et al.* 2024), and SetBERT (ours). Our work is highlighted in bold.

Model	Single-sequence processing	Sample-level processing	Raw sample processing	Pre-trainable
DNABERT	✓			✓
DNABERT 2	✓			✓
DNABERT-S	✓			✓
MetaBERTa	✓			✓
HyenaDNA	✓			✓
SetQuence/SetOmic	✓	✓		
glm2	✓			✓
Scorpio	✓			✓
**SetBERT**	✓	✓	✓	✓

Since taxonomy labels are required, we pre-train on synthetic versions of the four datasets. To produce these synthetic dataset versions, we follow a protocol similar to that of CAMISIM (Fritz *et al.* 2019), where we first compute a taxonomy profile of each of our samples and randomly generate synthetic samples comprised of SILVA sequences. More specifically, we assign taxonomy labels to all of the DNA sequences, and then replace each DNA sequence with a DNA sequence from SILVA corresponding to the assigned label. This ensures SetBERT is trained on ground-truth reference data using realistic taxonomy distributions. While it is known that a considerable portion of the taxa in an environment is unknown (Nasko *et al.* 2018), we rely on the robustness of SetBERT to generalize to these unknown taxa during inference (and other downstream tasks working with the actual raw data). We show that, like CAMISIM (Fritz *et al.* 2019), the taxonomy distributions of our synthetic sample data are log-normal in Supplementary Fig. S5, available as supplementary data at *Bioinformatics* online, and are composed of a large variety of genera in Supplementary Fig. S4, available as supplementary data at *Bioinformatics* online. To pre-train SetBERT, we produced synthetic versions of our datasets using labels assigned by our own top-down DNABERT taxonomy model described in Section 2.4.

We pre-trained SetBERT using the Adam optimizer with a learning rate of 1e−4 until convergence using a batch size of 3. A batch is generated by randomly selecting synthetic samples with replacement, and then drawing *n* = 1000 DNA sequences uniformly from each sample to produce subsamples. Sequences within the subsamples are randomly trimmed on either end to 150 bp in length, and ambiguous bases are augmented according to standard IUPAC codes. Full training details are provided in the Supplementary Materials, available as supplementary data at *Bioinformatics* online. Once SetBERT is pre-trained, it can be quickly fine-tuned for stream tasks of any kind such as regression, single/multi-label classification, etc. Table 1 provides high-level feature comparisons between SetBERT and other models in the current literature.

### 2.4 Fine-tuning: classification and explainability

We fine-tuned two different sample-level binary classification models starting from our pre-trained SetBERT model using the Hopland and SFD datasets to predict the class of particular samples. Samples from the Hopland data were gathered from two different soil regions: bulk and rhizosphere. We construct a binary classifier to predict the soil sample region by attaching a single dense layer with one output neuron and the sigmoid activation function. As we are updating the parameters of the pre-trained model during fine-tuning, we follow prior works (Devlin *et al.* 2019, Dosovitskiy *et al.* 2021) and predict directly from the transformed [CLS] token as opposed to averaging the transformed sequence embeddings (Nguyen *et al.* 2023, Zhou *et al.* 2024b). For the SFD dataset, we grouped the data into positive/negative-presence of *Ophidiomyces ophidiicola*. We built a binary classifier in the same manner as described with the Hopland data. Each of these classification models was trained until convergence. The full training details are described in the Supplementary Materials, available as supplementary data at *Bioinformatics* online.

After performing the classification tasks, we analyse the attention scores produced by the models to give us an indication as to which sequences are the most critical in the model’s classification process (i.e. explain the reason behind its predictions). We employ an adaptation of the *self-attention attribution* method by Hao *et al.* (2021) to produce attribution scores for each of the inputs. Additional implementation details and hyperparameter settings are provided in the Supplementary Materials, available as supplementary data at *Bioinformatics* online.

### 2.5 Fine-tuning: taxonomic assignment

In order to predict taxonomy with high accuracy, the model should take into account the hierarchical nature of taxonomy labels. BERTax is the current state-of-the-art deep learning architecture for taxonomic classification built on a custom BERT model with a hierarchical classification head (Mock *et al.* 2021).The head predicts a taxonomy label for each of the possible ranks, where all previous rank predictions affect the lower rank predictions. One down side to the BERTax architecture is that the number of parameters grows exponentially as the depth of the hierarchy increases. Furthermore, we argue that taxa do not need to be weighted based on unrelated higher-level taxa (siblings of parents). For these reasons, we propose the *top-down* classification head visualized in Supplementary Fig. S3, available as supplementary data at *Bioinformatics* online. Given a DNA sequence-level embedding, it is passed through a dense layer for each of the possible ranks, where each dense layer contains a neuron for each possible taxonomy label for that rank. Before computing the probabilities, the predicted logits for each of the taxa at the higher ranks are added to all subsequent child taxon logits resulting from the lower rank predictions. This allows the confidence of taxonomic ranks to properly weight their corresponding children. The final logits for each rank are then passed through a softmax to obtain probability distributions. For the SILVA dataset, the top-down architecture contains two orders-of-magnitude fewer parameters than BERTax while still including a hierarchical inductive bias.

We fine-tuned three different DNABERT models for taxonomy prediction, each with a different classification head. One model utilizes the BERTax head, another with our top-down head, and a final naive head to test the benefit of having a hierarchical classification head. This naive classification head is comprised of a single dense layer with a softmax activation function to predict the lowest-level taxonomic rank directly from the sequence embedding. During evaluation for each of the different heads, the label with the highest probability is chosen, and the confidence for each of the parent ranks is computed by summing across other probabilities that are under the corresponding hierarchy.

As SetBERT operates on entire samples, ground-truth samples again need to be available for fine-tuning and evaluation. We produce a synthetic version of each experimental dataset using the three deep learning taxonomy models (i.e. naive, BERTax, and top-down) fine-tuned from DNABERT and QIIME 2’s *feature classifier plugin* (Bokulich *et al.* 2018) as described previously in the SetBERT Pre-training section, resulting in four different versions of each dataset. Synthetic samples can then be produced using different taxonomy distributions and models can be evaluated on distributions produced by other models.

We fine-tune SetBERT (top-down) and evaluate all models across the four synthetic dataset versions. For each sample, 10 subsamples comprised of 10 000 randomly-drawn sequences trimmed to 150 bp are generated. The models then assign taxonomy labels to each sequence down to the genus level. This is repeated for each version of synthetic samples (i.e. synthetic sample distributions generated by QIIME 2, Naive, BERTax, and Top-down generated). The complete details are provided in the Supplementary Materials, available as supplementary data at *Bioinformatics* online.

## 3 Results

### 3.1 Entire samples can be represented as vectors

BERT-style models have the ability to represent entire inputs in a single contextualized vector representation. For natural language models, this means that whole sentences or paragraphs can be represented with a single vector. SetBERT is able to take advantage of this ability to represent entire samples with a single vector representation. This is a powerful feature of SetBERT in that it allows one to very quickly measure the similarity of samples simply using vector similarity functions such as cosine similarity or euclidean distance.

**Figure 2.**
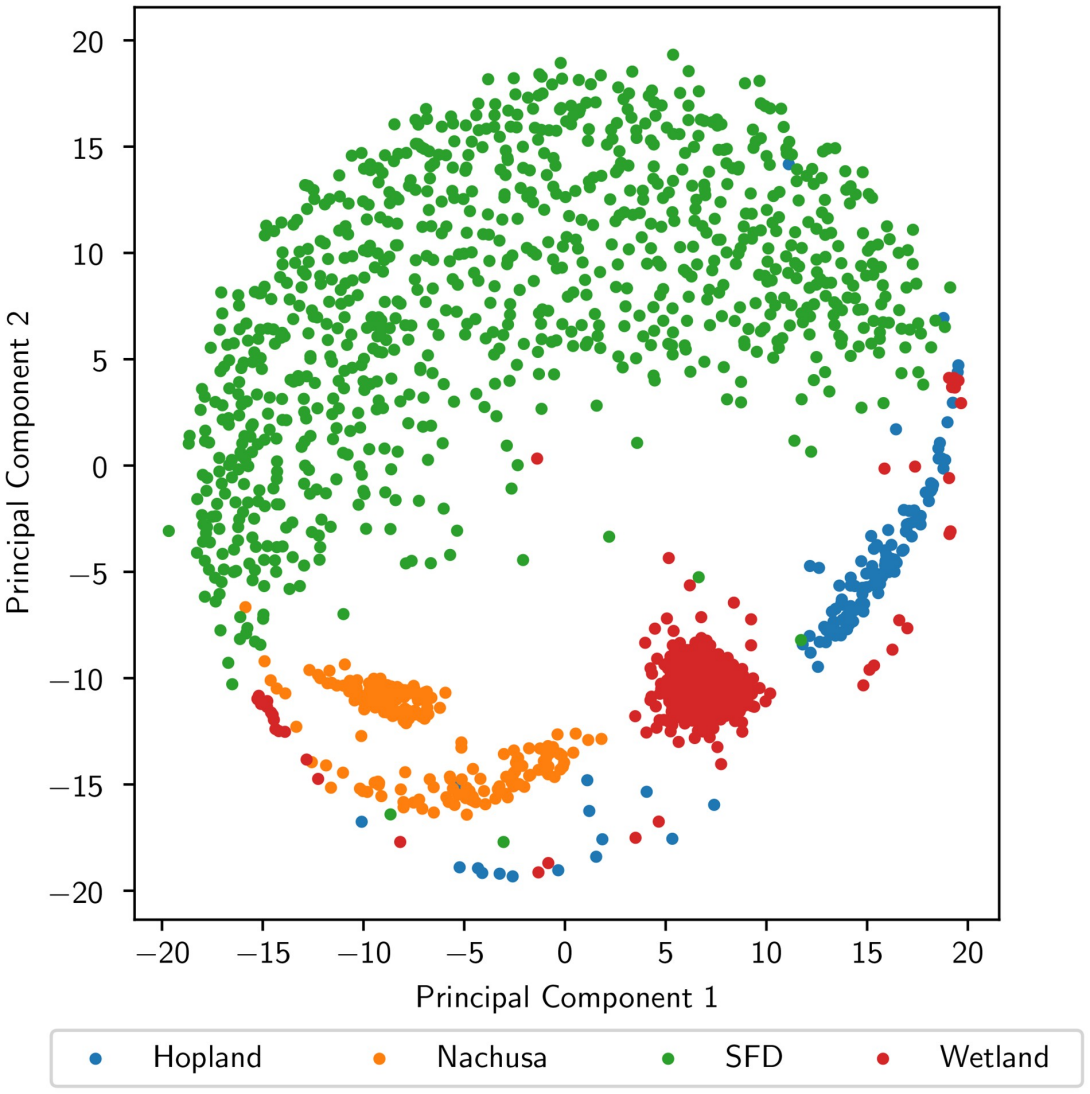
An MDS plot of pre-trained SetBERT sample embeddings grouped by dataset. Each point represents a subsample comprised of 1000 sequences drawn with replacement from a corresponding sample. Points closer to each other are more similar, while points further apart are more dissimilar.

**Figure 3.**
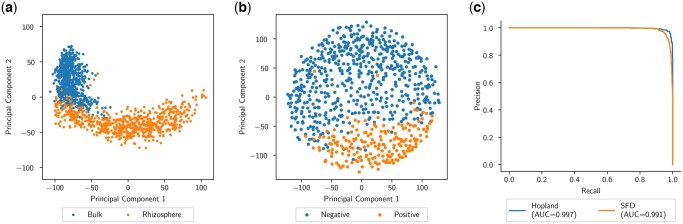
The resulting fine-tuned SetBERT sample embeddings plotted via MDS and precision/recall curves with associated area under the precision/recall curve (AUC) scores for the classifiers. Each point in the MDS plots represents a subsample of 1000 sequences drawn from a corresponding sample. The clear division of regions shown in the plot confirms that the model is able to accurately partition the samples in the latent space. The precision/recall curves indicate attainable precision/recall performance given a particular classification threshold. An experimenter may tweak this threshold to control for the measure they wish to be the most precise. (a) MDS of Hopland Embeddings. (b) MDS of SFD Embeddings. (c) Precision/Recall Curve.

To demonstrate this process, we compute latent-space embeddings of our real samples from the SetBERT models and plot them in two-dimensions using metric multi-dimensional scaling (MDS). Figure 2 is an MDS plot of the sample-level embeddings from the pre-trained SetBERT model. The similarity between samples is directly related to the distance between them where subsample points that are close together are more similar to each other than two points which are further apart. This plot shows a distinct division between the three soil datasets (i.e. Hopland, Nachusa, and Wetland) and the SFD dataset, where nearly all of the SFD subsample points reside on the opposite side of the latent-space. This result demonstrates that SetBERT was able to capture the similarities of the soil samples and recognize a distinction between soil and snake skin microbiomes. We can also see a clear separation between bulk/rhizosphere and disease state in Fig. 3a and b, respectively, from the fine-tuned binary classifiers’ sample-level embeddings. The performance of these classifiers is measured via precision and recall in Fig. 3c, where we obtained area under the precisions–recall curve (AUC) scores over 0.99. Most importantly, because all of these distinctions are captured in the MDS space using the real sample data as opposed to the synthetic data it was pre-trained on, our results suggest that SetBERT is robust enough to handle misclassified/misrepresented sequences in the synthetic sample data and continue to produce meaningful embeddings.

### 3.2 SetBERT’s predictions are explainable

**Figure 4.**
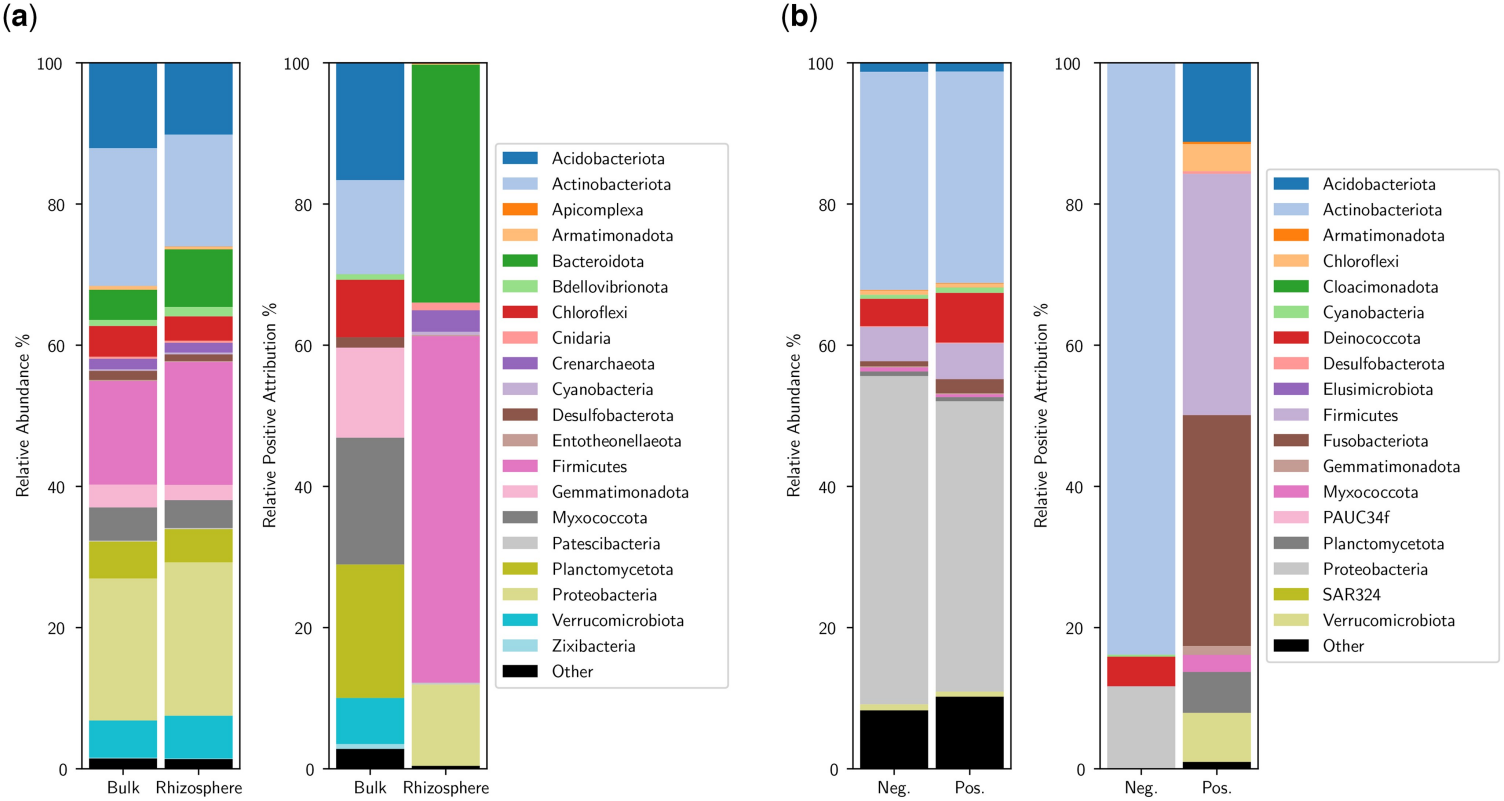
The top 10 taxa by sample class from the Hopland and SFD binary classifiers. For each classifier, the top 10 positive-scoring taxa are gathered in each class and merged into a single list without duplicates. The first pair of bars represent the relative abundance of the top scoring taxa in the sample class, while the second pair of bars represent the relative positive attribution score. A higher attribution score indicates that the presence of the taxonomy increases the likelihood of the model choosing the corresponding class. (a) Hopland. (b) SFD.

We demonstrate the critical taxa identification process by evaluating the Hopland and SFD classification models. For both datasets, 10 subsamples comprised of 1000 DNA sequences were drawn from each of the samples and evaluated to obtain attention attribution scores for the input DNA sequences. Each DNA sequence was then assigned a taxonomic label and scores were aggregated by taxa via summation. The top 10 scoring phyla by attention attribution score were extracted from each sample class and plotted in Fig. 4 via relative abundance and relative positive attribution. These two distributions side-by-side show that there is not a direct correlation between taxonomy abundance and class prediction (e.g. relative abundance of Deinococcota and Proteobacteria in SFD samples), indicating that the model is able to leverage the interactions between the microorganisms to make more informed decisions.

Analysing the relative positive attribution distribution of the Hopland classifier in Fig. 4a, we find that the model identifies Bacteroidota, Firmicutes, and Proteobacteria as significant contributors for rhizosphere classification. These microbial classes are known to be abundant in the rhizosphere of a large number of plant species (Mendes *et al.* 2013). Proteobacteria and Bacteroidota exhibit a preference for carbon-rich environments, resulting in their increased abundance within the rhizosphere, driven by their high metabolic rates and fast growth (Ling *et al.* 2022). Likewise, our model also identifies Actinobacteriota as an indicator for bulk soil, where it is known to be more prevalent compared to the rhizosphere (Shi *et al.* 2015). This group of bacteria is equipped with a large set of polymer-degrading enzymes that enable it to successfully survive in soil environments where plant-exuded carbon is not available (Zhalnina *et al.* 2018, 2022). Thus, we can confirm that the taxa scored the highest by the model are indeed biologically relevant.

It is important to note that Apicomplexa and Cnidaria were identified among the top contributors; however, these microbes should not be present. Since these samples were evaluated without any pre-processing, the identification of these microorganisms was most likely due to the presence of contaminant sequences. However, the relative attribution score for these sequences is extremely low and can safely be ignored by only acknowledging sequences with a minimum or significant score threshold.

Looking at the relative attribution of phyla for SFD classification in Fig. 4b, we find that Actinobacteriota were the most useful for negative classification. Streptomyces is found in this phylum and known to produce many secondary metabolites and antimicrobial compounds (Barka *et al.* 2016). Examining the relative abundance reveals that there is little difference between the positive and negative samples; yet, it is labeled as one of the most critical taxa for negative class identification in the positive attribution scores. This is again due to the fact that abundance on its own is not directly correlated with positive/negative. Rather, it is the difference in interactions between the Actinobacteriota and other present bacteria that drive negative classification.

### 3.3 Context improves taxonomic assignment accuracy

One of the limitations of many taxonomy models is that they assign labels to each DNA sequence independently. Without the context (other DNA sequences/organisms present), each assignment is essentially a shot-in-the-dark prediction. This can make it significantly more difficult to resolve ambiguity. Researchers have made use of weighted classifiers that were trained on particular kinds of habitats in order to boost classification accuracy (Kaehler *et al.* 2019). The downside of this approach is that an experimenter must choose the appropriate classifier for their samples. SetBERT captures the interactions between the input sequences allowing it to make taxonomic predictions in a contextualized manner fully autonomously. Since we aim to present SetBERT as a generalized prediction model, we only compare our predictions with unweighted classifiers.

**Figure 5.**
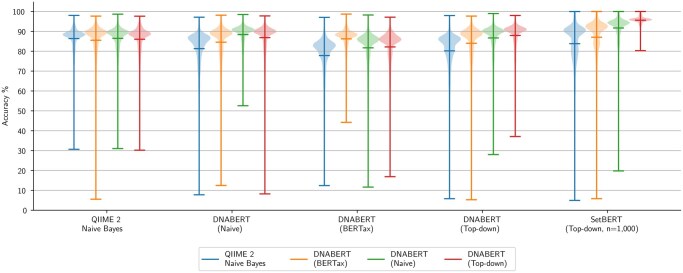
Genus-level taxonomy classification accuracy by model. Each group of violins represents the classification accuracy of a particular model. Each violin represents the distribution of average classification accuracies across all subsamples. The color of the violin indicates the taxonomy model used to produce the synthetic samples.

The models are evaluated across all of the subsamples generated from each of the four synthetic dataset versions. The results are visualized via a violin plot in Fig. 5 and the numerical results are shown in Table 2. Each violin represents the distribution of average classification accuracy for genus-level prediction by subsample across all datasets. The color of the violin corresponds to the model used to generate the synthetic samples (i.e. synthetic sample version) as a means of measuring and ruling out predictive bias. The QIIME 2 and DNABERT models assign taxonomy labels independently, while SetBERT assigns taxonomy labels with a window size of *n*, where *n* sequences are evaluated simultaneously.

**Table 2.** The median taxonomy classification accuracy and 95% confidence interval of each model by synthetic dataset version.

Model	QIIME 2 Naive Bayes	DNABERT (Naive)	DNABERT (BERTax)	DNABERT (Top-down)
QIIME 2 Naive Bayes	0.8808 ± 0.0004	0.8869 ± 0.0005	0.8888 ± 0.0007	0.8834 ± 0.0005
DNABERT (Naive)	0.8471 ± 0.0008	0.9039 ± 0.0004	0.8851 ± 0.0009	0.8947 ± 0.0005
DNABERT (BERTax)	0.8159 ± 0.0008	0.8532 ± 0.0007	0.8793 ± 0.0004	0.8549 ± 0.0007
DNABERT (Top-down)	0.8344 ± 0.0010	0.8952 ± 0.0006	0.8829 ± 0.0009	0.9047 ± 0.0005
DNABERT (Top-down, Deep)	0.8281 ± 0.0010	0.8937 ± 0.0006	0.8815 ± 0.0010	0.8952 ± 0.0007
SetBERT (Top-down, *n* = 1)	0.8178 ± 0.0015	0.8776 ± 0.0008	0.8494 ± 0.0013	0.8976 ± 0.0005
SetBERT (Top-down, *n* = 1000)	0.8814 ± 0.0013	0.9390 ± 0.0005	0.9148 ± 0.0010	0.9587 ± 0.0001
SetBERT (Top-down, *n* = 10 000)	**0.8820** ± **0.0013**	**0.9394** ± **0.0005**	**0.9154** ± **0.0010**	**0.9590** ± **0.0001**

The row corresponds with the model being evaluated and the column indicates the synthetic dataset version. Bold values highlight the model with the highest median classification accuracy of samples from the corresponding synthetic dataset version.

Examining Fig. 5, the single-sequence models are able to classify taxa with comparable performance, while SetBERT is able to push the predictive accuracy even further. It is important to point out that the models’ performances are affected by the sample versions as they may contain distributions more in favor of the model’s predictive bias. A model will more accurately classify samples with taxonomy distributions that it, itself, produced. This can be observed for each of the DNABERT models in Fig. 5. Even in these cases, however, SetBERT outperforms all other approaches measured in this work.

**Figure 6.**
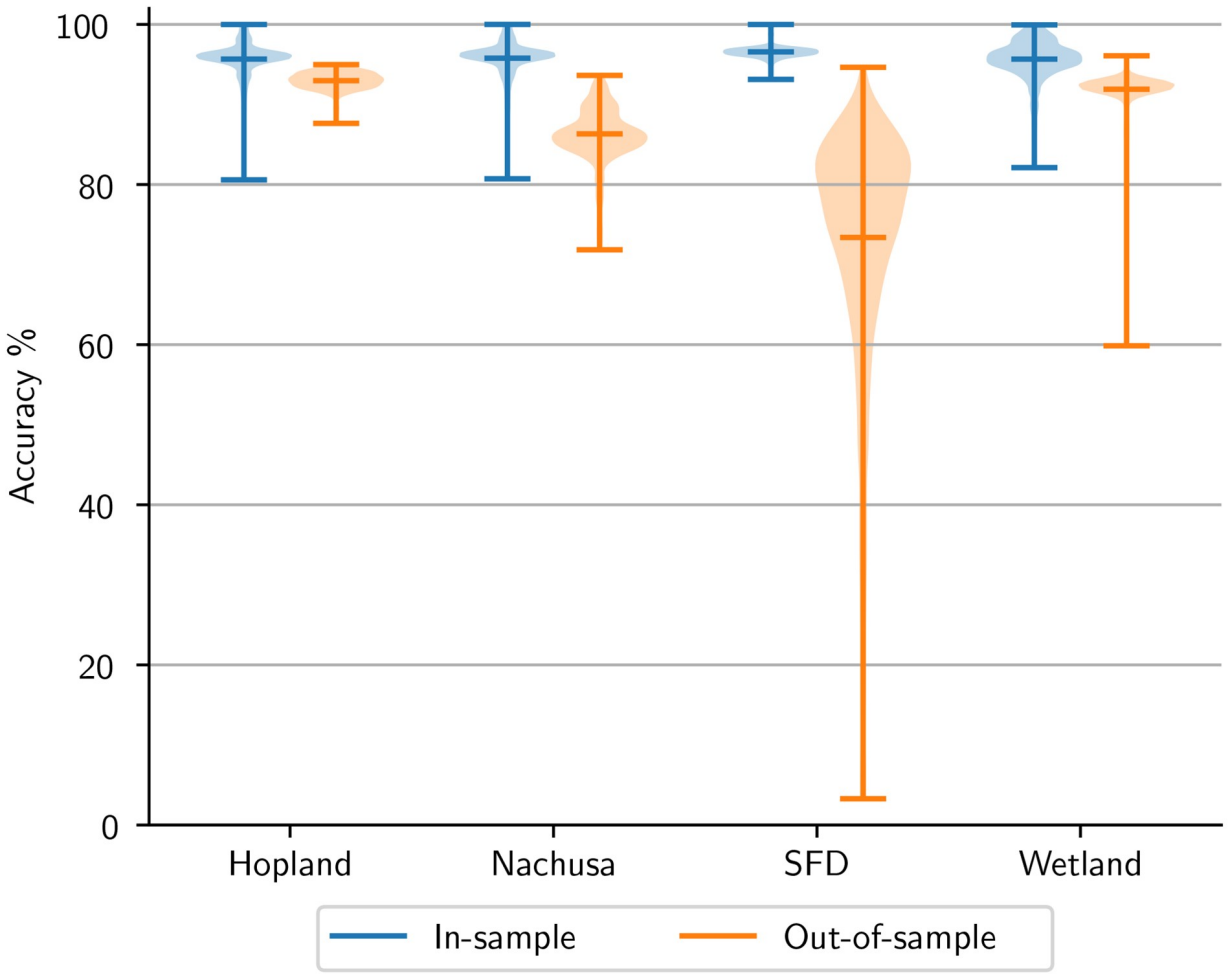
The genus-level taxonomy classification accuracy of datasets left out of training. Each dataset group contains two violins: in-sample and out-of-sample. The label corresponds with the dataset that was left out of the training data. The in-sample violins correspond to the classification accuracy of random subsamples generated from the remaining three datasets, while the out-of-sample violins correspond to the classification accuracy of random subsamples generated from the held-out dataset.

While the median classification accuracy is relatively high for each of the models presented, there are still subsamples that can result in very low classification accuracy as indicated by the lower tails in the violin plots. This is due to the model’s inability to disambiguate the sequences, resulting in misclassification. This result demonstrates the key limitation of the single-sequence models that SetBERT sets out to solve. It can be noted that SetBERT is also prone to these same misclassifications when evaluated on sample versions produced by the other architectures. However, this makes sense as SetBERT is leveraging the learned taxonomy distributions to disambiguate the sequences. Samples produced by the other architectures can yield taxonomy distributions that significantly deviate from the samples that SetBERT was trained on. In these cases, SetBERT cannot utilize sequence interactions as reliably. However, misclassifications are significantly reduced when SetBERT is evaluated on the kinds of distributions that it was exposed to during training as shown by the loss of the violin tails in Figs 5 and 6.

It is important to consider the fact that the SetBERT model is twice as deep as the DNABERT models and, therefore, contains many more learnable parameters. This subjects SetBERT to a potential advantage simply from the number of parameters available. In order to demonstrate that the performance of SetBERT is achieved by leveraging the sequence interactions rather than an increase in model size, we control for the learnable parameter count by presenting a top-down DNABERT model (DNABERT (Top-down, Deep)) with twice the number of transformer blocks. Since SetBERT uses DNABERT to embed the DNA sequences, SetBERT contains 16 transformer blocks in its pipeline compared to the eight transformer blocks of the DNABERT classification models. By constructing a DNABERT model with 16 transformer blocks, the models effectively have the same learnable capacity. The results are visualized in Supplementary Fig. S4, available as supplementary data at *Bioinformatics* online. Examining the numerical results in Table 2 (Top-down, Deep), we find that the additional transformer blocks in the deep DNABERT model do not yield any increase in performance when compared to the standard DNABERT model.

Finally, we evaluate SetBERT with various window sizes to determine how the number of DNA sequences provided at once impact classification performance. We evaluate SetBERT with window sizes of n=1 (i.e. single-sequence evaluation), *n* = 1000 and *n* = 10 000. The results of these evaluations are shown in Table 2 and compared in Supplementary Fig. S6, available as supplementary data at *Bioinformatics* online. By reducing the window size to n=1, the model can no longer leverage any sequence interactions to help disambiguate sequence predictions. As expected, the decreased performance is in line with the DNABERT prediction models. However, due to the bias of sequence distributions in the samples, the model behaves more like a weighted classifier, yielding both positive and negative impacts to performance for particular samples which is captured in the violin plots. It is only when the window size allows for parsing many sequences at once (i.e. *n* = 1000 and *n* = 10 000) that we see a significant increase in performance. This result confirms that SetBERT’s increase in performance is due to its ability to make contextualized predictions via the captured sequence interactions. Furthermore, we also show that SetBERT can extrapolate to higher set sizes (*n* = 10 000) during inference to push classification accuracy even further. This is possible because the additional memory overhead limiting subsample size during model training can instead be leveraged at inference time to process larger subsamples.

### 3.4 SetBERT generalizes to similar habitats

Language models based on BERT are known to be highly generalizable (Devlin *et al.* 2019). As more training data is provided, the generalization of the model typically continues to improve. Since SetBERT relies on sequence interactions to make predictions, the performance of the model is highly dependent upon the habitats/biomes observed during training. We briefly explore SetBERT’s ability to generalize to other habitats/biomes by pre-training and fine-tuning four independent SetBERT models, each holding out a corresponding dataset from its training data.

We evaluated each of the four models across all four datasets in the same manner as described in the previous subsection and plotted the results in Fig. 6. Each violin group corresponds to the dataset that was discarded during training. The *In-sample* violins represent the genus-level taxonomy prediction accuracy distribution for subsamples generated from the training datasets, while *Out-of-sample* represents the accuracy distribution for subsamples generated from the held-out testing dataset. In this plot, we observe that SetBERT is able to generalize well to the Hopland, Nachusa, and Wetland datasets when they are left out of training. However, there is a significant performance reduction when generalizing to SFD samples. This matches our expectations as Hopland, Nachusa, and Wetland are all soil-based ecosystems while the SFD dataset is not. Because SetBERT has seen other soil-based ecosystems during training when leaving out either Hopland, Nachusa, or Wetland, it is able to transfer knowledge to other data of similar modality and leverage sequence interactions to continue to perform well for those out-of-domain samples. However, since the SFD dataset is the only snake skin ecosystem, it would be unlikely to expect any model to understand what a snake skin microbiome looks like in order to leverage neighbor interactions.

## 4 Discussion

In this work, we proposed SetBERT: a novel and highly generalizable deep learning approach for analysing HTS samples. We found that SetBERT is highly performant in that it is able to reliably leverage DNA sequence interactions to make more accurate predictions. By pre-training on a variety of datasets across different biomes, the model is able to generalize to similar environments. Furthermore, we were able to mine attention scores for the relevant taxa used for the model’s predictions as well as confirm their biological significance in existing literature.

SetBERT was pre-trained on a limited number of datasets for this work, mostly comprised of soil with the additional snake fungal disease dataset. Moreover, this work focused on 16S data as this is what our collaborators were sequencing. As was verified in the taxonomic classification results, SetBERT is able to generalize well to other microbiomes of similar type since it is familiar with those microbial communities. Deep learning models such as BERT that have been trained on vast amounts of data have been shown to have excellent generalization and extrapolation to unseen examples. Likewise, the performance of transformer models has been shown to improve with additional data. For these reasons, we believe that giving SetBERT more high-quality and diverse data from different ecosystems could significantly improve its performance and allow this tool to scale and generalize to many different habitats. This could be repeated for different sequencing regions to produce a broader set of reusable models. Doing so will require careful curation of datasets across a wide variety of ecosystems to be effective.

Another important note regarding SetBERT is its ability to work with raw sequencing data. Deep learning approaches are appealing, not only in their ability to solve hard tasks, but also for their ability to work with raw or minimally-processed data. Sequencing runs typically require post-processing in order to remove contaminants, chimeras, etc. Moreover, most other models and statistical techniques also require extensive pre-processing to ensure meaningful results. Even when careful, unintentional pre-processing bias may impact final results. Given enough raw training data, SetBERT might be able to learn to ignore contaminants and chimeras on its own without any guide from the experimenter and account for errors that occur within sequences using the knowledge of other sequences present in the sample.

During the development of SetBERT, other notable DNA sequence embedding models have since been published (Nguyen *et al.* 2023, Refahi *et al.* 2023, Zhou *et al.* 2024a, 2024b). These models have been shown to produce improved DNA sequence embeddings in different aspects, such as capturing species awareness, and other various characteristics. These pre-trained models could potentially provide a notable performance boost to SetBERT if used instead of our pre-trained DNABERT model. Since we update the parameters of these sequence embedding models during the pre-training and fine-tuning of SetBERT, it is likely that these models would lose track of these characteristics. Not to mention that SetBERT could also be trained up without any pre-training of the DNA sequence embedding models at all. However, it could also be that the embeddings produced by these pre-trained models are conditioned well enough to not require parameter updates during training. For these reasons, we proceeded to complete our experiments with the original DNABERT architecture as it was enough to bootstrap SetBERT’s learning process.

Our results showed that SetBERT was able to extrapolate to sample sizes an order of magnitude larger than what was experienced during training to maintain and even improve performance. The limitation of the sample sizes during training and inference is primarily limited by the computational resources available. During training, the primary memory bottleneck is the gradients of the DNA sequence embedding models as we allow their parameters to be updated. During inference, DNA sequences can be embedded ahead of time, thus SetBERT’s transformer architecture becomes the bottleneck with a quadratic space complexity of $O(n^{2})$. It is possible to improve both of these inefficiencies at the cost of performance that was not investigated in this work. Firstly, replacing our pre-trained DNABERT model with one of the pre-trained DNA sequence embedding models mentioned previously could provide sufficient enough embeddings that SetBERT can be trained effectively while holding the embedding model’s parameters constant. This would significantly improve training scalability at the cost of embedding expressiveness. Secondly, it is also possible to improve the space complexity of SetBERT by replacing the standard *Set Attention Blocks* with Set Transformer’s *Induced Set Attention Blocks* to achieve a linear space complexity of O(nm) where *m* is a tunable hyperparameter. This change would allow SetBERT to scale significantly during training and inference at the cost of performance. However, even without any of these additional optimizations, SetBERT was able to scale a whole order of magnitude during inference without any additional computing resources.

SetBERT is available as source code as well as a plugin to the QIIME 2 platform. We supply pre-trained SetBERT QIIME 2 artifacts. Through the plugin, an experimenter may also pre-train their own SetBERT model tuned for the kinds of tasks they wish to solve through the QIIME 2 Pipeline. SetBERT can be fine-tuned for taxonomic classification as well as for general classification. It also includes the tools to analyse attention scores via attention attribution as performed in the Hopland/SFD datasets.

## Supplementary Material

btaf370_Supplementary_Data

## Data Availability

The data for the Hopland, Nachusa, Wetland datasets are available through GenBank Sequence Read Archive (SRA) under accession numbers 1139056, 1154704, and 1129551, respectively. The SFD data are available through in GenBank SRA under accession numbers 1114724 and 1114659. All source code is publicly available on Github (https://github.com/DLii-Research/setbert). Tutorial notebooks and documentation for training custom SetBERT models are available. The q2-deepdna QIIME 2 plugin for utilizing the latest pre-trained SetBERT model is available on PyPI, and the source code is available on Github at https://github.com/DLii-Research/q2-deepdna.
