# Supplementary material for: SetBERT: the deep learning platform for contextualized embeddings and explainable predictions from high-throughput sequencing
Source: Bioinformatics. 2025 Jun 25;41(7):btaf370. doi: 10.1093/bioinformatics/btaf370 (PMC12245400; doi:10.1093/bioinformatics/btaf370)
Supplement: btaf370_Supplementary_Data [file btaf370_supplementary_data.pdf]

Supplementary materials for "SetBERT: the deep learning platform for contextualized embeddings and explainable predictions from high-throughput sequencing "

David W. Ludwig II, Christopher Guptil, N. Reed Alexander, Kateryna Zhalnina, Edi M-L Wipf, Albina Khasanova, Nicholas A. Barber, Wesley Swingley, Donald M. Walker, Joshua L. Phillips

1 Data Gathering Methods

1.1 Hopland microbial community: experimental design, sample collection and processing

Rhizosphere samples and corresponding bulk soil samples were collected in four replicates from 15 cm deep 'wedges'. The samples were harvested at four time points during active vegetation growth (March 2020), flowering (April 2020), the beginning of senescence (May 2020), and at the end of the dry season (October 2020). The experimental design included the addition of 59 g m^{-2} of urea and reduced water availability.

Soil microbial DNA was isolated from 0.25 g of soil, using the DNeasy PowerSoil Pro Kit (Qiagen), following the manufacturer's protocol. The V4 regions of 16S rRNA genes in DNA samples were amplified with the 515F and 806R primer set (Parada et al. (2016); Caporaso et al. (2011)) and sequenced on an Illumina MiSeq platform (2x151 bp run) at the Argonne National Laboratory.

1.2 Prairie soil samples from Nachusa Grasslands

Sampling took place in spring (April or May), summer (July), and fall (September or October) in 2015, 2016, 2017, and 2020. In 2020, one additional restored prairie site (planted in 2016) was sampled. During sampling, each site was visited and 5 subsamples of soil from the top 5cm were collected from an area approximately 1 ha in size. The subsamples from each site were pooled, homogenized, and stored on ice until transportation to a lab the same day for storage at -80°C.

Soil microbial DNA was isolated from 0.25 g of soil, using the DNeasy PowerSoil Pro Kit (Qiagen), following the manufacturer's protocol. The V4 regions of 16S rRNA genes in DNA samples were amplified with the 515F and 806R primer set (Parada et al. (2016); Caporaso et al. (2011)) and sequenced on an Illumina MiSeq platform (2x151 bp run) at Rush University Genomics and Microbiome Core Facility..

1.3 Snake fungal disease skin swabs

Samples for this study were processed at the Walker Lab at Middle Tennessee State University (MTSU). DNA was isolated from snake skin swabs using independent DNeasy PowerSoil HTP 96 kits (Qiagen), following the manufacturer's standard protocol. The V4 variable region of the 16S rRNA gene was amplified using the 515F and 806R primer pair (Kozich et al. (2013)) and sequenced on an Illumina MiSeq using V2 chemistry (2 x 250 bp pair-end reads) in the Walker Lab at MTSU. Bioinformatics analysis was performed in mothur (Schloss et al. (2009)), and sequences were clustered into operational taxonomic units (OTUs) at 97% sequence similarity (Walker et al. (2019)). The R package decontam was used to flag contaminating sequences, and OTUs identified as contaminants were removed from the respective databases.

1.4 Restored federal wetland soils

The samples for this study were processed at the Walker Lab at MTSU. DNA was isolated from wetland soil samples (0.25 g of homogenized soil slurry) using independent DNeasy PowerSoil HTP 96 kits (Qiagen), following the manufacturer's standard protocol. The V4 variable region of the 16S rRNA gene was amplified using the 515F and 806R primer pair (Kozich et al. (2013)) and sequenced on an Illumina MiSeq using V2 chemistry (2 x 250 bp pair-end reads) in the Walker Lab at MTSU. Bioinformatics analysis was performed in mothur (Schloss et al. (2009)), and sequences were clustered into operational taxonomic units (OTUs) at 97% sequence similarity (Walker et al. (2019)). The R package decontam was used to flag contaminating sequences, and OTUs identified as contaminants were removed from the respective databases.

2 Architecture Implementation and Training Details

2.1 Transformer Architecture Implementation

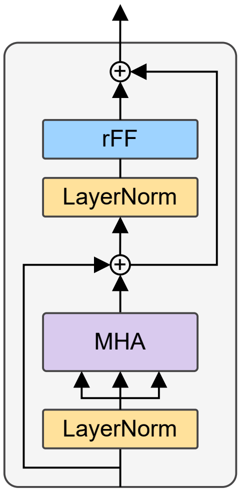

Figure 1: The transformer architecture used in both the standard transformer implementation (Vaswani et al. (2017)) for DNABERT and the Set Attention Block (SAB) implementation (Lee et al. (2019)) used in SetBERT. LayerNorm is layer normalization by Ba et al. (2016) and rFF is a 2-layer row-wise feed-forward network with the GeLU activation function (Hendrycks and Gimpel (2023)) between them.

The primary component of a transformer block is the multi-head attention (MHA) layer (Vaswani et al. (2017)). MHA compares a set of queries, Q, to a set of keys, K, to weight a set of values, V, corresponding to each key. Q, K, and V are each comprised of d_k, d_k, and d_v-dimensional vectors respectively packed into matrices. Formally, this represents a mechanism for learning to focus attention (i.e. scaled dot-product attention) on relevant data features and is defined as:

$$\text{AttentionScore}(Q, K) = \text{softmax}\left(\frac{QK^\top}{\sqrt{d_k}}\right) \tag{1}$$

$$\text{Attention}(Q, K, V) = \text{AttentionScore}(Q, K)V \tag{2}$$

Multi-head attention is performed by computing and concatenating the results of attention using different learned linear projections of the inputs like so:

$$\begin{aligned} \text{MHA}(Q, K, V) &= \text{Concat}(\text{head}_i, ..., \text{head}_h)W^O \\ \text{where head}_i &= \text{Attention}(QW_i^Q, KW_i^K, VW_i^V) \end{aligned} \tag{3}$$

Here, $W_i^Q \in \mathbb{R}^{d_{\text{model}} \times d_k}$, $W_i^K \in \mathbb{R}^{d_{\text{model}} \times d_k}$, $W_i^V \in \mathbb{R}^{d_{\text{model}} \times d_v}$, and $W_i^O \in \mathbb{R}^{hd_v \times d_{\text{model}}}$ are learned parameter matrices (Vaswani et al. (2017)). Our implementation incorporates the change in the order

of layer-normalization (Ba et al. (2016)) that occurs within the transformer block by (Xiong et al. (2020)) which has been shown to improve training stability/performance by providing a better highway for gradients to flow back to prior layers. The formal definition of our transformer architecture is as follows:

$$\begin{aligned} \text{Transformer}(M) &= H + \text{rFF}(\text{LayerNorm}(H)) \\ \text{where } H &= M + \text{MHA}(M_{\text{ln}}, M_{\text{ln}}, M_{\text{ln}}), \\ \text{and } M_{\text{ln}} &= \text{LayerNorm}(M) \end{aligned} \tag{4}$$

where rFF is a 2-layer row-wise feed-forward network with the GeLU activation function between them (Hendrycks and Gimpel (2023)).

We construct the model following the methodologies of the Set Transformer (ST) framework (Lee et al. (2019)) in order to make the model compatible with unstructured, set-based data. The framework notes that the MHA mechanism within the transformer is naturally permutation-equivariant, making it ideal for handling unstructured data. Models that leverage input token order rely on explicit injection of position information by the experimenter either through the form of learned embeddings or precomputed encodings.

2.2 Attention Attribution Implementation

Attention attribution for a particular head is computed using the Riemann approximation described in the equation below:

$$\begin{aligned} A^l &= [A^l_1, ..., A^l_{|h|}] \\ \text{Attr}_h(A^l) &= \frac{A^l_h}{m} \odot \sum_{k=1}^{m} \frac{\partial F(\frac{k}{m} A^l)}{\partial A^l_h} \end{aligned} \tag{5}$$

where h is an attention head for a transformer layer and $A^l_h = \text{AttentionScore}(Q^l_h, K^l_h)$ is the attention score matrix for head h. Like the paper, we chose the number of integration steps to be $m = 20$. The attribution scores for each layer are obtained by aggregating the scores across the attention heads via summation like so:

$$S^l = \sum_{h=1}^{|h|} \text{Attr}_h(A^l) \tag{6}$$

We compute the attribution score for individual DNA sequences by aggregating the rows in the attention attribution scores scaled by the largest positive attribution value:

$$S_j = \sum_{l=1}^{|l|} \frac{\sum_{i=1}^{n} s^l_{i,j}}{\max(S^l)} \tag{7}$$

where j corresponds with the input DNA sequence index and $s^l_{i,j}$ is the attention attribution score of the interaction between input sequences i and j at layer l. Positive attribution scores indicate that the sequence is beneficial for predicting the particular class, and vice-versa.

2.3 DNABERT Implementation for High-throughput Sequencing

The original DNABERT model was built for whole-genome sequencing in mind; however, we are working with short-read gene fragments. This allows us to make several simplifications to make the model more fit for HTS data. First, we held the input sequence length constant to fit our read lengths, allowing us to remove the special padding token ([PAD]). Next, we continue to leave out the next-sentence/sequence prediction pre-training task (as per the BERT pre-training regime) since we are working with gene-fragments that span multiple microorganisms, removing the requirement for a separation token ([SEP]). Lastly, we employ relative-position encodings (Shaw et al. (2018); Huang et al. (2020)) rather than absolute-position encodings as we found them to embed sequences more consistently with regard to their alignment/shift, consistent with other NLP transformer-based models.

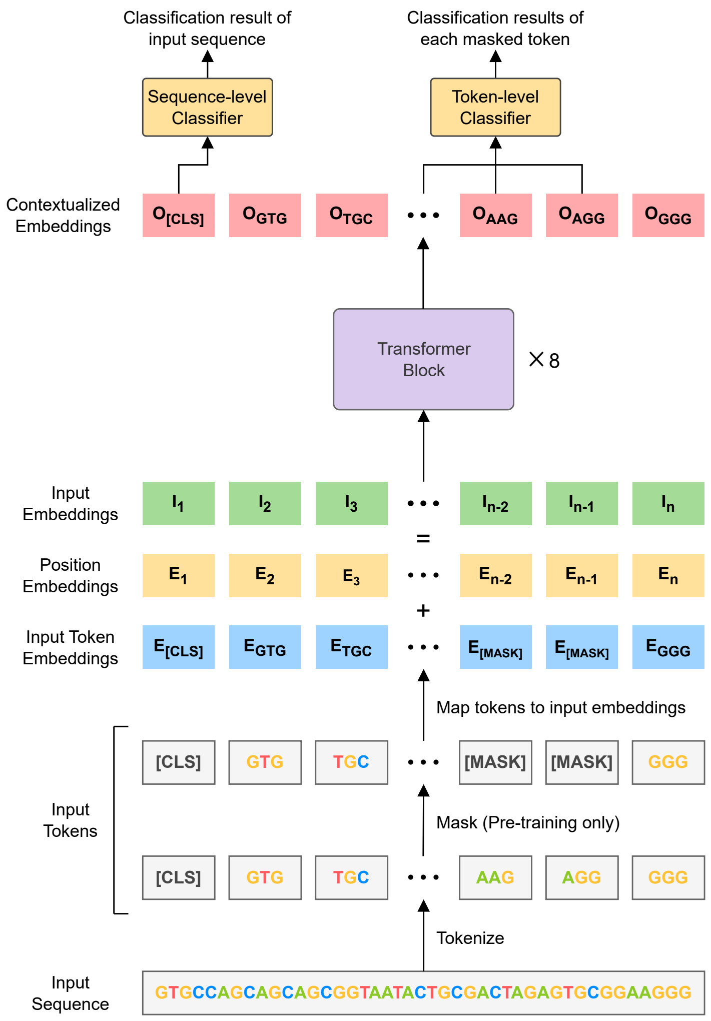

Figure 2: Our DNABERT model used for sequence-level embeddings. The sequence is first split into overlapping 3-mers. During pre-training, a random contiguous block comprised of 15% of the 3-mers is masked. Lastly, the tokens are mapped to corresponding learned input embeddings, position embeddings are added via summation, and everything is passed through a stack of transformer blocks. The output [CLS] embedding is a contextualized vector representation of the entire sequence, and each output 3-mer embedding is a representation of its corresponding input 3-mer in context to the rest of the sequence.

2.4 Top-down Architecture

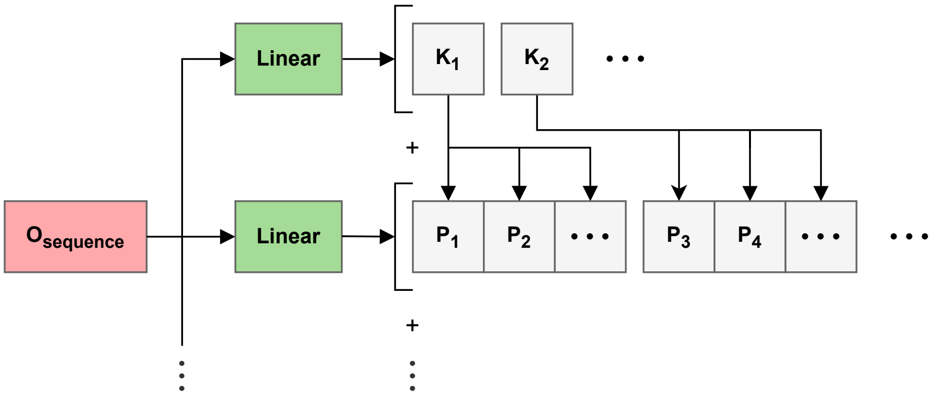

Figure 3: Our top-down taxonomy prediction architecture. The logits for each taxonomy rank are first predicted from the input DNA sequence embedding independently. The predicted rank logits are weighted by adding their immediate corresponding parent logits. This diagram demonstrates this process via the kingdom and phylum ranks.

3 Training Details & Hyperparameter Settings

3.1 DNABERT Pre-training

We pre-trained our DNABERT model on the SILVA v138.1 NR99 SSU 16S/18S dataset (Pruesse et al. (2007)) with sequences trimmed to the V3-V4 region following the procedures outlined by QIIME 2 using the standard 515F/806R primer pair (Kozich et al. (2013); Quast et al. (2013); Bokulich et al. (2018); Robeson et al. (2021)). It accepts DNA sequences of 150 bp in length as input, yielding 64-dimensional sequence embeddings. Sequences longer than 150 bp are randomly truncated on either end to 150 bp. Ambiguous bases are replaced with corresponding concrete bases drawn uniformly based on the standard IUPAC codes. The model is comprised of 8 transformer layers, each with 8 attention heads. It was trained for 200,000 steps with the Adam optimizer and a batch size of 256. As per the DNABERT paper (Ji et al. (2021)), a learning rate schedule was employed where it increased from 0 to 1e-4 for the first 10,000 steps, and linearly decreased back to 0 until the final step.

3.2 SetBERT Pre-training

We pre-trained SetBERT using the synthetic datasets produced by the top-down DNABERT taxonomy model for 60,000 steps with a batch size of 3. A batch is comprised of subsamples of samples drawn uniformly at random across all of the datasets. Each subsample is created by drawing $n_{\text{sample}} = 1,000$ sequences within the corresponding sample uniformly with replacement and then randomly truncating each sequence to 150 bp by trimming either end, as well as randomly augmenting any ambiguous bases. The Adam optimizer was used with a fixed learning rate of 1e-4.

3.3 Synthetic Dataset Taxonomy Distributions

For each of our datasets, we performed taxonomic assignment with four different models: QIIME 2, DNABERT (Naive), DNABERT (BERTax) (Mock et al. (2021)), and DNABERT (Top-down). We then produced synthetic versions of each our datasets for each taxonomy model by replacing the raw reads with corresponding reads from the SILVA dataset trimmed to the 16S region as described in the main manuscript. We then created 10 samples comprised of 10,000 sequences from each synthetic sample available across all synthetic versions of the dataset. Figure 4 provides boxplots indicating the number of unique genera per sample for each model and dataset. Likewise, Figure 5 provides histograms showing that the unique genera frequency generally follows a lognormal distribution as expected (Fritz et al. (2019)).

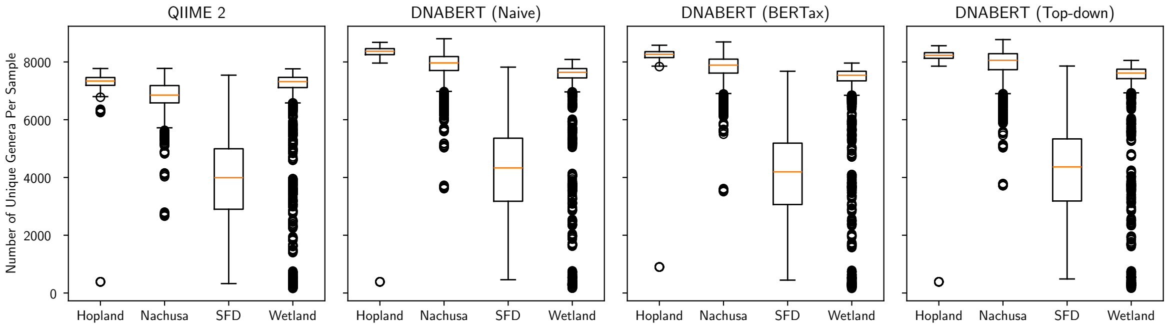

Figure 4: The number of unique genera in each evaluation sample by dataset for each model. Each evaluation sample is comprised of 10,000 random SILVA sequences guided by the taxonomy profile of their real sample counterparts.

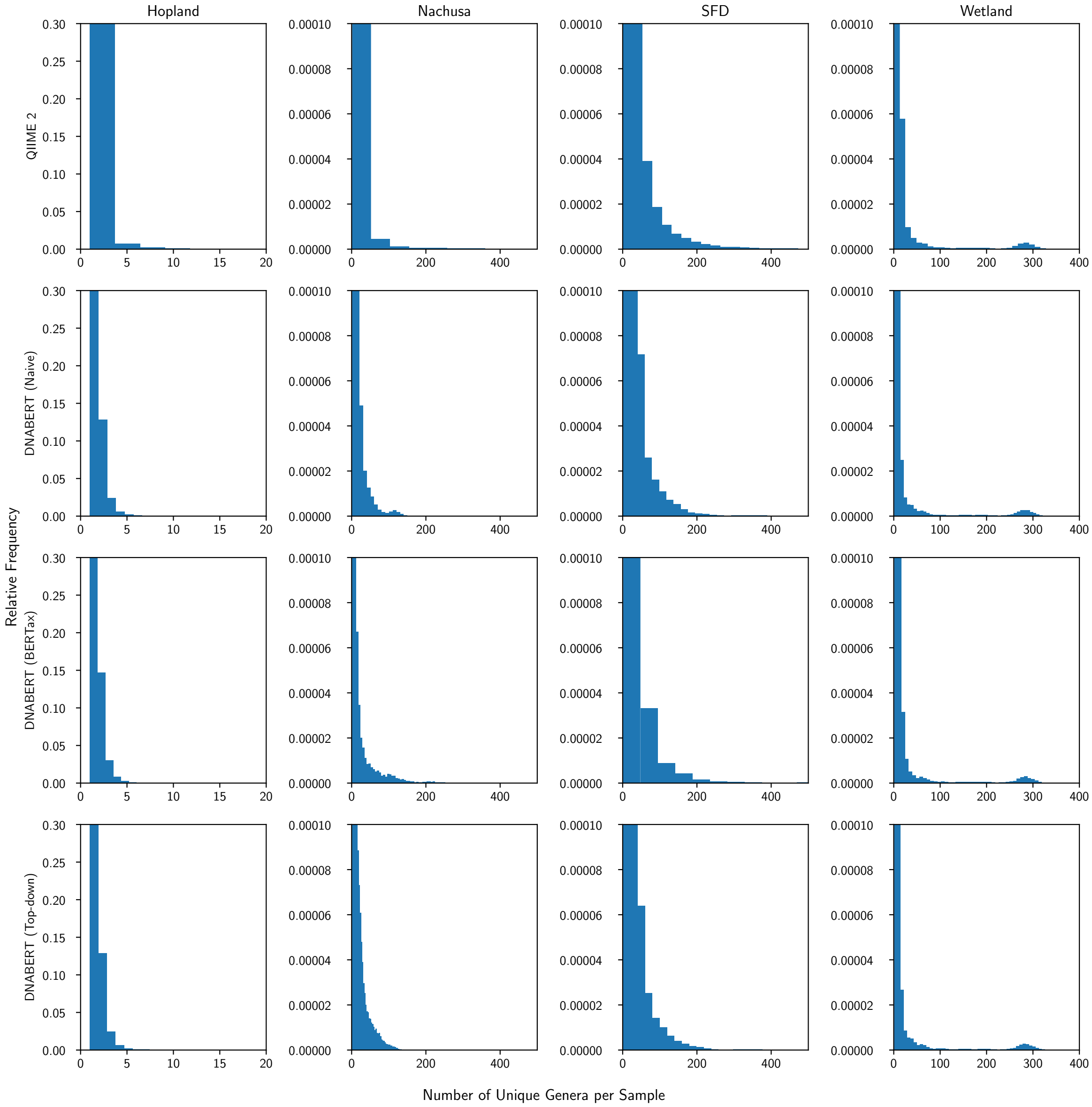

Figure 5: The density of unique genera per sample for the evaluation samples by dataset and taxonomy model. Due to the high number of unique taxa per sample, the y-axis has been cropped in order to visualize the shape of the frequency distribution.

3.4 Fine-tuning DNABERT and SetBERT Models for Taxonomic Classification

We fine-tune three independent DNABERT models starting from the same pre-trained instance used in the SetBERT kernel, each using one of the corresponding taxonomy classification heads described above. Each model is trained for 200,000 steps with a batch size of 256 sequences using the Adam optimizer with a constant learning rate of 1e-4.

We fine-tune SetBERT for taxonomic classification using the top-down classification head architecture. It is trained on random subsamples of 1,000 sequences using the synthetic datasets whose distributions were produced by the DNABERT (top-down). It was trained for 50,000 steps with a batch size of 3 using the Adam optimizer with a learning rate of 1e-4.

3.5 Fine-tuning SetBERT for Bulk/Rhizosphere & SFD Positive/Negative Classification

We fine-tuned two different binary classification models built on our pre-trained SetBERT model using the Hopland and SFD datasets. The models were trained with a batch size of 3 until convergence using the Adam optimizer with a learning rate of 1e-4 on subsamples comprised of 1,000 sequences generated from the synthetic datasets.

4 Additional Results Figures

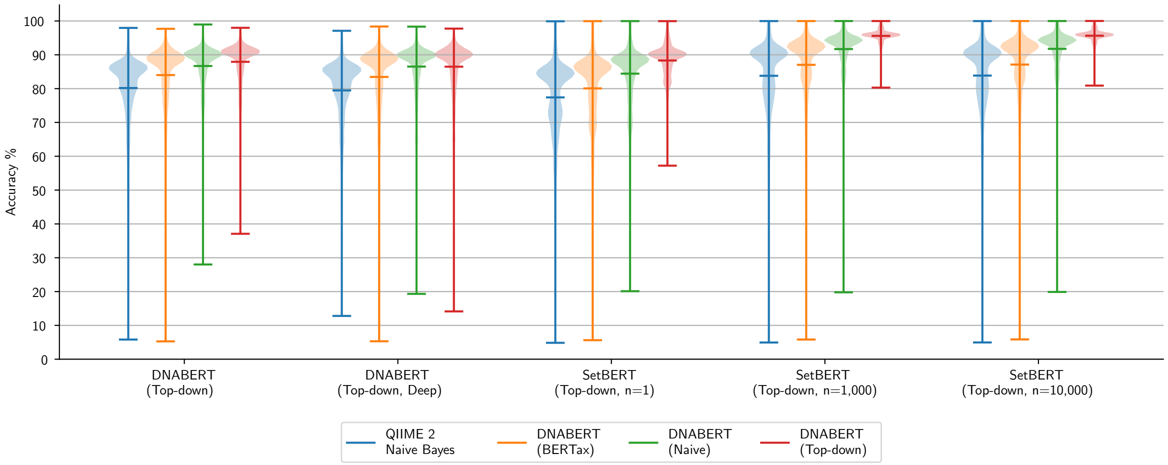

Figure 6: Taxonomy classification accuracy controlling for model parameter count and the subsample size for SetBERT. Each violin represents the genus-level classification accuracy distributions for synthetic subsamples comprised of 10,000 sequences across the Hopland, Nachusa, SFD, and Wetland datasets.

References

Ba, J. L., Kiros, J. R., and Hinton, G. E. (2016). Layer Normalization. Number: arXiv:1607.06450 arXiv:1607.06450 [cs, stat].

Bokulich, N. A., Kaehler, B. D., Rideout, J. R., Dillon, M., Bolyen, E., Knight, R., Huttley, G. A., and Gregory Caporaso, J. (2018). Optimizing taxonomic classification of marker-gene amplicon sequences with QIIME 2's q2-feature-classifier plugin. *Microbiome*, 6(1):90.

Caporaso, J. G., Lauber, C. L., Walters, W. A., Berg-Lyons, D., Lozupone, C. A., Turnbaugh, P. J., Fierer, N., and Knight, R. (2011). Global patterns of 16S rRNA diversity at a depth of millions of sequences per sample. *Proceedings of the National Academy of Sciences*, 108(supplement_1):4516–4522.

Fritz, A., Hofmann, P., Majda, S., Dahms, E., Dröge, J., Fiedler, J., Lesker, T. R., Belmann, P., DeMaere, M. Z., Darling, A. E., Sczyrba, A., Bremges, A., and McHardy, A. C. (2019). CAMISIM: simulating metagenomes and microbial communities. *Microbiome*, 7(1):17.

Hendrycks, D. and Gimpel, K. (2023). Gaussian Error Linear Units (GELUs). arXiv:1606.08415 [cs].

Huang, Z., Liang, D., Xu, P., and Xiang, B. (2020). Improve Transformer Models with Better Relative Position Embeddings. Number: arXiv:2009.13658 arXiv:2009.13658 [cs].

Ji, Y., Zhou, Z., Liu, H., and Davuluri, R. V. (2021). DNABERT: pre-trained Bidirectional Encoder Representations from Transformers model for DNA-language in genome. *Bioinformatics*, 37(15):2112–2120.

Kozich, J. J., Westcott, S. L., Baxter, N. T., Highlander, S. K., and Schloss, P. D. (2013). Development of a Dual-Index Sequencing Strategy and Curation Pipeline for Analyzing Amplicon Sequence Data on the MiSeq Illumina Sequencing Platform. *Applied and Environmental Microbiology*, 79(17):5112–5120.

Lee, J., Lee, Y., Kim, J., Kosiorek, A. R., Choi, S., and Teh, Y. W. (2019). Set Transformer: A Framework for Attention-based Permutation-Invariant Neural Networks. *arXiv:1810.00825 [cs, stat]*. arXiv: 1810.00825.

Mock, F., Kretschmer, F., Kriese, A., Böcker, S., and Marz, M. (2021). BERTax: taxonomic classification of DNA sequences with Deep Neural Networks. Pages: 2021.07.09.451778 Section: New Results.

Parada, A. E., Needham, D. M., and Fuhrman, J. A. (2016). Every base matters: assessing small subunit rRNA primers for marine microbiomes with mock communities, time series and global field samples. *Environmental Microbiology*, 18(5):1403–1414.

Pruesse, E., Quast, C., Knittel, K., Fuchs, B. M., Ludwig, W., Peplies, J., and Glöckner, F. O. (2007). SILVA: a comprehensive online resource for quality checked and aligned ribosomal RNA sequence data compatible with ARB. *Nucleic Acids Research*, 35(21):7188–7196.

Quast, C., Pruesse, E., Yilmaz, P., Gerken, J., Schweer, T., Yarza, P., Peplies, J., and Glöckner, F. O. (2013). The SILVA ribosomal RNA gene database project: improved data processing and web-based tools. *Nucleic Acids Research*, 41(D1):D590–D596.

Robeson, M. S., O'Rourke, D. R., Kaehler, B. D., Ziemski, M., Dillon, M. R., Foster, J. T., and Bokulich, N. A. (2021). RESCRIPt: Reproducible sequence taxonomy reference database management. *PLOS Computational Biology*, 17(11):e1009581.

Schloss, P. D., Westcott, S. L., Ryabin, T., Hall, J. R., Hartmann, M., Hollister, E. B., Lesniewski, R. A., Oakley, B. B., Parks, D. H., Robinson, C. J., Sahl, J. W., Stres, B., Thallinger, G. G., Van Horn, D. J., and Weber, C. F. (2009). Introducing mothur: open-source, platform-independent, community-supported software for describing and comparing microbial communities. *Applied and Environmental Microbiology*, 75(23):7537–7541.

Shaw, P., Uszkoreit, J., and Vaswani, A. (2018). Self-Attention with Relative Position Representations. *arXiv:1803.02155 [cs]*. arXiv: 1803.02155.

Vaswani, A., Shazeer, N., Parmar, N., Uszkoreit, J., Jones, L., Gomez, A. N., Kaiser, , and Polosukhin, I. (2017). Attention is All you Need. In *Advances in Neural Information Processing Systems*, volume 30. Curran Associates, Inc.

Walker, D. M., Leys, J. E., Grisnik, M., Grajal-Puche, A., Murray, C. M., and Allender, M. C. (2019). Variability in snake skin microbial assemblages across spatial scales and disease states. *The ISME Journal*, 13(9):2209–2222.

Xiong, R., Yang, Y., He, D., Zheng, K., Zheng, S., Xing, C., Zhang, H., Lan, Y., Wang, L., and Liu, T.-Y. (2020). On Layer Normalization in the Transformer Architecture. arXiv:2002.04745 [cs, stat].
